# Fabrication of Polyimide/Aluminum Nitride Composites and Wafer Channel Filling via Direct Ink Writing

**DOI:** 10.3390/nano16110681

**Published:** 2026-05-31

**Authors:** Junjie Xiao, Qingjie Shan, Zhoulong Xu, Zhouping Yin, Bin Xie, Hao Wu

**Affiliations:** 1 State Key Laboratory of Intelligent Manufacturing Equipment and Technology, Huazhong University of Science and Technology, Wuhan 430074, China; xj_jie@hust.edu.cn (J.X.); qingjie0128@hust.edu.cn (Q.S.); yinzhp@hust.edu.cn (Z.Y.); 2 Wuhan Chiplet Technology Co., Ltd., Wuhan 430074, China; xuzhoulong@chiplettech.com

**Keywords:** PI/AlN composite ink, coefficient of thermal expansion, direct ink writing (DIW) technology, thermal stability, wafer channels filling

## Abstract

The emergence of three-dimensional heterogeneous integration (3D HI) has pushed forward the development of chip-to-wafer (C2W) hybrid bonding technology. To mitigate stress concentration during thermal annealing and wafer thinning processes of C2W bonding, a direct ink writing (DIW)-based 3D printing approach was proposed to fill the channel between two adjacent chips on the bonded wafer (i.e., wafer channels). A composite slurry consisting of polyimide (PI) as base material and aluminum nitride (AlN) nanoparticles as fillers was prepared. Through surface chemical modification and ultrasonic treatment, the slurry featured uniform filler dispersion (with particle size less than 1 μm) and adequate viscosity (3327 mPa·s), which fits the 3D printing process. The cured film demonstrated superior thermal stability and mechanical properties compared with pure PI, with a coefficient of thermal expansion (CTE) of 4.97 ppm/K, which matched that of silicon-based materials and exhibited excellent bonding. This approach provides a cost-effective and efficient alternative to chemical vapor deposition (CVD) techniques for filling wafer channels.

## 1. Introduction

With the advancements in artificial intelligence (AI) and high-performance computing (HPC), Moore’s Law is approaching its physical limits, prompting the development of three-dimensional heterogeneous integration (3D HI) technologies as a new frontier in packaging [1,2,3]. Wafer-level packaging, a critical method for achieving 3D HI, frequently encounters challenges such as wafer warpage during the chip-to-wafer (C2W) bonding process [4,5,6]. To mitigate stress concentration issues arising from wafer thinning and bonding, materials with a coefficient of thermal expansion (CTE) like that of silicon are employed to fill the channel between two adjacent chips on the bonded wafer (i.e., wafer channels). Currently, high-density plasma-enhanced chemical vapor deposition (PECVD) is the prevalent technique for wafer channels filling [7,8]. However, this approach is hindered by its low efficiency, high cost, and stringent environmental requirements, which impede its utilization in the wafer channels filling application.

As an alternative approach, the direct ink writing (DIW) technique is a 3D printing technology based on a material extrusion process. Owing to its wide range of available materials, simple operation, easy molding and other advantages, DIW technique is currently recognized as one of the most widely used 3D printing methods [9,10]. However, the major obstacle for implementing the DIW technique to fill the wafer channels is the preparation of a composite slurry that features thermal–mechanical properties that match those of silicon-based wafers, and adequate viscosity that fits the DIW process. However, there is a tradeoff between the requirements of ‘silicon-like’ thermal–mechanical properties and the ‘ink-like’ viscous property. Polymer organic materials with good temperature resistance and flow properties are a suitable material for this solution. Polyimide (PI) has been widely studied due to its excellent comprehensive performance, especially in the field of electronic packaging [11]. However, its coefficient of thermal expansion significantly diverges from that of silicon, potentially resulting in wafer warpage and interfacial delamination [12,13,14]. Recent studies have explored various inorganic nanoparticles to balance the thermal and mechanical properties of PI-based composites [15,16,17]. Nevertheless, issues such as nanoparticle agglomeration and sedimentation often yield excessively large particles and insufficient filling concentrations, limiting their applicability for wafer channel filling.

To address these challenges, we proposed a composite slurry that consists of polyimide (PI) as polymer matrix and aluminum nitride (AlN) ceramic nanoparticles as fillers. Silane coupling agent was applied to functionalize the surface of AlN nanoparticles, thereby lowering their surface energy and mitigating agglomeration. The modified nanoparticles were prepared through dispersant evaporation followed by ball milling. During the mixing process with PI, the AlN nanoparticles were initially dispersed in the solvent N-methyl-2-pyrrolidone (NMP), and then incrementally combined with a high-viscosity PI solution in multiple stages. Then, the DIW technique was developed to extrude the as-prepared PI/AlN composite slurry. The slurry extrusion speed was controlled by pneumatics. The filling parameters were set before filling, and continuously adjusted according to the real-time monitored filling situation during the printing process to ensure the filling accuracy. A certain thickness of slurry is pre-filled at the intersection of the channels to avoid defects due to insufficient slurry at the intersection during the curing process. The experimental results show that the slurry can be smoothly extruded from a 30 μm glass needle and fill the entire wafer channels without voids. After thermal curing, the resulting film showed a low coefficient of thermal expansion (4.97 ppm/K), high thermal conductivity (2.514 W/m·K), and robust mechanical stability. This work provides a new approach toward an efficient and cost-effective wafer channel filling process.

## 2. Materials and Methods

### 2.1. Materials

To facilitate the effective filling of wafer channels through DIW technology, it is imperative that the composite ink demonstrates superior flowability, uniformity, and exceptional thermal stability. Additionally, the coefficient of thermal expansion of the composite ink must be compatible with that of the silicon substrate. Polyimide was selected as the matrix material due to its outstanding thermal resistance and favorable rheological properties (U-imide-BH, UNITIKA Ltd., Osaka, Japan), which features a 26% concentration and an absolute viscosity of 5000 mPa·s at 30 °C. For the inclusion of nano-ceramic particles, it is essential that these particles exhibit excellent dispersion and compatibility with the polyimide solution. Aluminum nitride (AlN) particles were selected due to their CTE of 4.5 ppm/K, which closely matches the CTE of silicon (3.5–4 ppm/K), and their well dispersion properties in the polyimide solution [18,19,20]. Nano-sized AlN particles with a diameter of less than 100 nm (Sigma-Aldrich, St. Louis, MO, USA) were adopted. The choice of solvent for the polyimide was N-methyl-2-pyrrolidone (NMP), which has a high boiling point and facilitates optimal processing. A silane coupling agent was employed to enhance the interface bonding between the inorganic nanoparticles and the organic polyimide matrix [21]. The selected silane coupling agent, γ-aminopropyltriethoxysilane (KH550), features reactive groups that form covalent bonds with hydroxyl groups on the inorganic surface and hydrogen or covalent bonds with the organic polyimide, thereby improving the dispersion and compatibility of the nanoparticles within the matrix [22].

### 2.2. Preparation Process of Composite Ink

**Figure 1:**
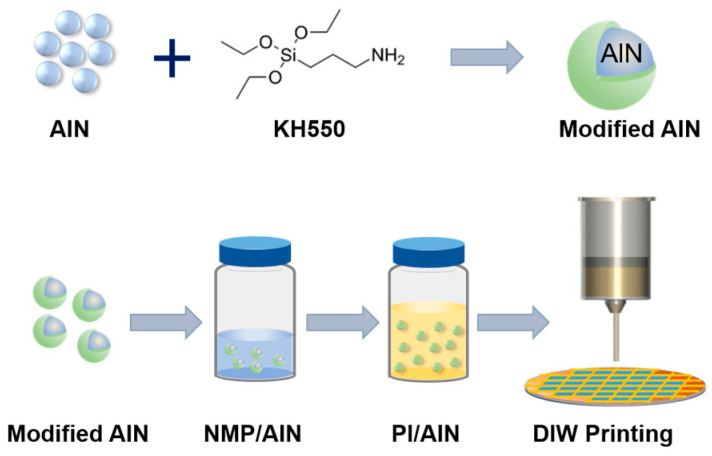
Schematic showing the preparation process of PI/AlN composite ink.

The preparation process of the composite slurry is illustrated in Figure 1. First, surface modification of the nano-aluminum nitride (AlN) particles is performed. The nano-AlN particles are uniformly dispersed in anhydrous ethanol, and silane coupling agent KH550 is added. This mixture undergoes ultrasonic treatment again to ensure thorough reaction between the nano-AlN particles and the coupling agent, thereby achieving interface modification of the nano-AlN particles. Subsequently, the modified nano-AlN particles are obtained by evaporating the anhydrous ethanol at 80 °C.

After ball-milling the nano-AlN particles, they are mixed with polyimide. During the mixing process, the nano-AlN particles are dispersed in the polyimide solvent, NMP, and then the high-viscosity polyimide solution is slowly added in multiple steps. Stirring and ultrasonic treatment are employed to ensure uniform mixing, resulting in PI/AlN composite ink. Specifically, the ultrasonic treatment was conducted for 10 min with a frequency of 40 kHz. The ball milling process was conducted at a rotational speed of 300 rpm for a duration of no less than 1 h. Depending on the viscosity of the mixture, the stirring rate was progressively increased from 500 rpm to 1200 rpm, with each stirring step lasting more than 30 min. This general procedure was used to prepare polyimide composite ink with aluminum nitride contents of 0, 10 wt%, 15 wt%, 20 wt%, 25 wt%, and 30 wt%. Using DIW technology, the composite slurry was filled into wafer channels using a glass needle with an inner diameter of 30 μm.

## 3. Results

### 3.1. Printability of Composite Ink

#### 3.1.1. Analysis of AlN Modification

**Figure 2:**
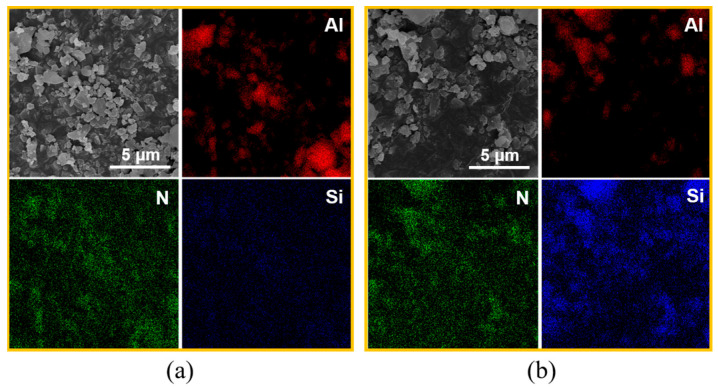
SEM and EDS images of (**a**) unmodified and (**b**) modified nano-AlN particles.

Figure 2 shows the Scanning Electron Microscopy (SEM) and Energy Dispersive Spectroscopy (EDS) images of the unmodified (Figure 2a) and modified nano-AlN particles (Figure 2b). It can be observed that a protective layer forms on the surface of the modified nano-AlN particles, with the silane coupling agent uniformly distributed on the surface. The distribution of the Si element further supports this observation.

**Figure 3:**
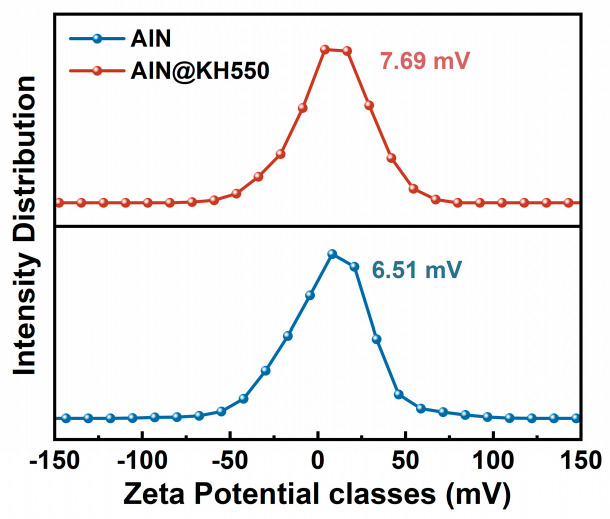
Zeta potential characterization of the AlN particles with/without surface modification.

Surface modification effectively reduces particle agglomeration, improves the dispersibility of nano-AlN particles, and enhances their adhesion to the polyimide matrix. We also measured the zeta potential of the unmodified and modified nano-AlN particles under the same testing conditions, as shown in Figure 3. The results indicate that the zeta potential of the modified particles is significantly higher, suggesting better dispersibility after modification. It should be noted that the numerical value is related to the test conditions, and this result is achieved under the same test conditions.

#### 3.1.2. Analysis of Viscosity

**Figure 4:**
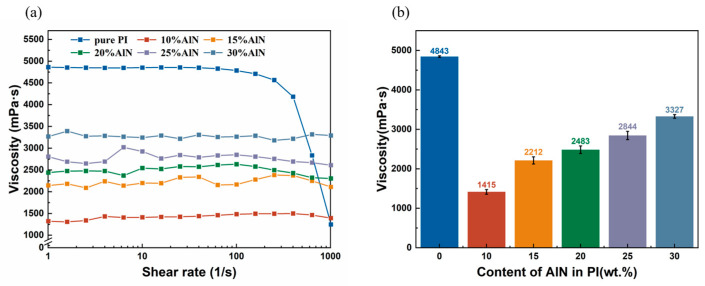
(**a**) Viscosity characterization of the composite slurry with different AlN contents; (**b**) Dependence of viscosity on AlN content (at 40 s^−1^ shear rate).

For successful application of DIW technology in filling wafer channels, the composite slurry must possess suitable viscosity. If the viscosity is too low, particles may settle, and flow rate control during extrusion becomes challenging, leading to potential overflow on the wafer surface. Conversely, if the viscosity is too high, it becomes difficult to form a uniformly dispersed system, which can cause needle clogging and other issues. Based on the preliminary experimental results and the DIW process limitations, the viscosity range of the material was selected. The viscosity range of 1000–5000 mPa·s for the composite slurry is suitable. We measured the viscosity of the prepared composite ink using a rheometer, with results shown in Figure 4. Compared with pure PI, which exhibits higher viscosity and shear-thinning behavior at elevated shear rates, the addition of NMP and nano-AlN particles resulted in a composite viscosity range of 1400–3500 mPa·s. It is worth noting that the composite slurry used for printing flows directly into the channels after extrusion and does not require self-supporting capability. Therefore, the composite slurry does not need to exhibit shear-thinning behavior. Practical printing tests confirmed that the viscosity was appropriate, allowing for stable and smooth extrusion.

#### 3.1.3. Solid Content Analysis

**Figure 5:**
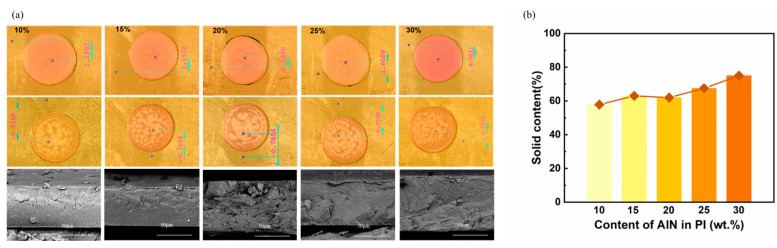
(**a**) Solid content test results and cross-sectional SEM images with different AlN contents; (**b**) dependence of solid content values on AlN content.

During the imidization process of the composite ink, solvent evaporation induces a certain degree of volume shrinkage, necessitating repeated filling and imidization steps. Consequently, the composite material must have a high-volume solid content to reduce the number of fillings and improve filling efficiency. The solid volume content of the composite ink was determined by measuring the wet film thickness before curing and the dry film thickness after curing using a non-contact film thickness gauge. As shown in Figure 5, the solid volume content of the composite ink increases significantly with the increase in AlN concentration. When the AlN content reaches 30%, the solid volume content can reach 75%, which can effectively reduce the number of fillings and enhance filling efficiency.

### 3.2. Compatibility of Composite Films

#### 3.2.1. Imidization Analysis

**Figure 6:**
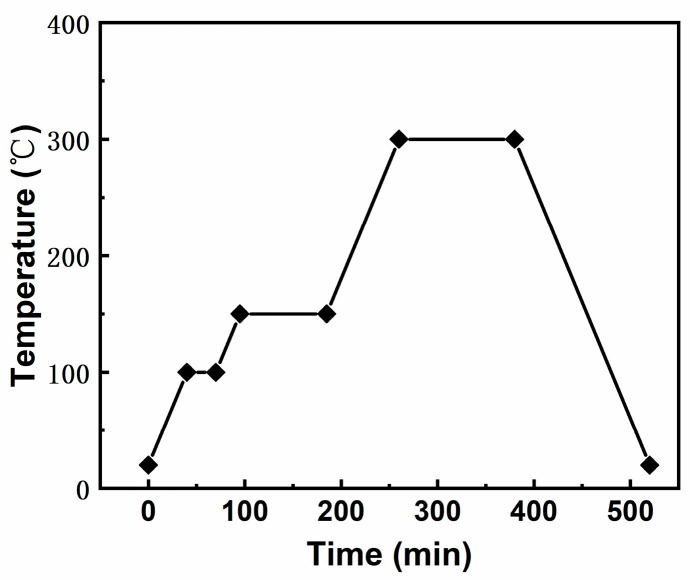
Temperature curve of the thermal imidization process.

The imidization process has a significant impact on the properties of polyimide composite films. It is crucial to slowly evaporate the solvent during the imidization process, since a rapid increase in temperature will lead to high internal stress, resulting in film cracking. Conversely, prolonged heating can cause film aging. In this study, a gradient imidization method was employed to cure the composite slurry. The temperature of the thermal imidization process is illustrated in Figure 6. During the wafer channel filling process, multiple filling and imidization steps are required. After each filling, the solvent was first evaporated at a low temperature, followed by subsequent printing and filling steps. Once the wafer channel was completely filled, the imidization process was performed using the gradient curing method.

#### 3.2.2. Particle Size and Dispersion

**Figure 7:**
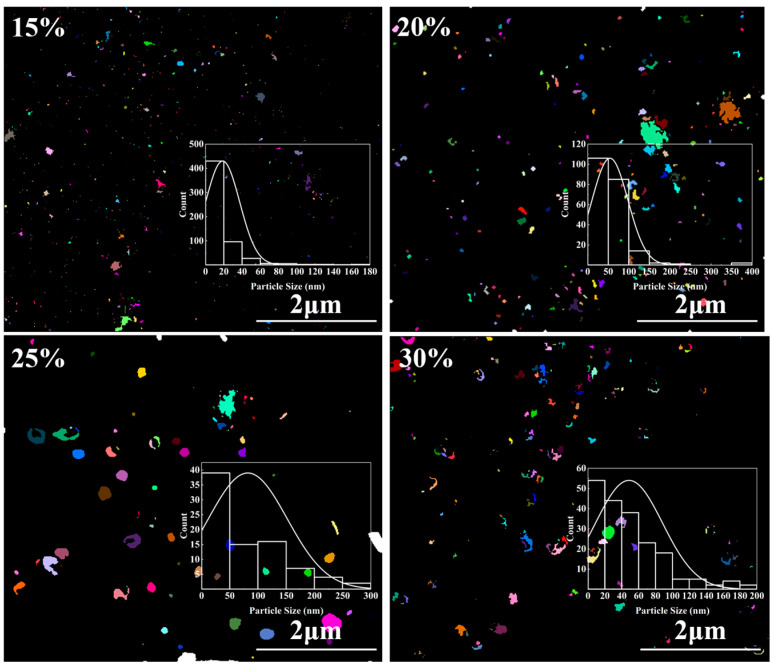
Processed SEM-based images showing the dispersion of AlN particles in composite films.

The size and uniformity of AlN particles within the composite slurry are crucial performance indicators for successful DIW filling of wafer channels. After curing the composite material into thin films, the spatial distribution of AlN particles was analyzed based on SEM micrographs, as shown in Figure 7. Through image-based particle segmentation and statistical analysis of multiple representative regions, the AlN particles were found to be well dispersed within the PI matrix without obvious agglomeration. The extracted particle features further indicate that most AlN particles are below 1 μm in size. Some particles are partially embedded within the polymer matrix, which may limit the exact boundary identification in SEM images. Nevertheless, the statistical results consistently confirm a submicron particle size distribution and a relatively homogeneous dispersion state.

#### 3.2.3. Structure of the AlN/PI Films

**Figure 8:**
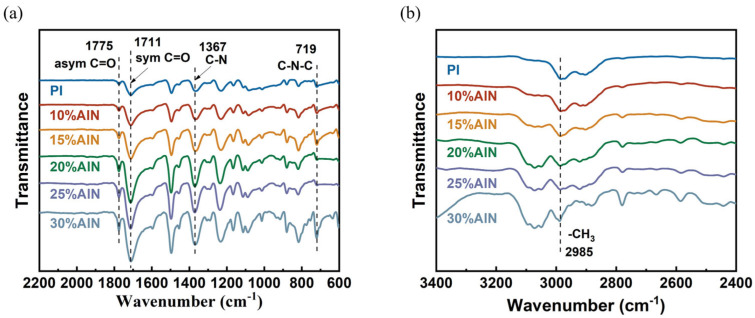
FT-IR spectra of the AlN/PI composite films: (**a**) 600–2200 cm^−1^; (**b**) 2400–3400 cm^−1^.

As shown in the FTIR spectrum in Figure 8, the absorption bands at 1775 cm^−1^ (symmetric stretching of imide carbonyl), 1711 cm^−1^ (asymmetric stretching of imide carbonyl), 1367 cm^−1^ (C-N stretching vibration), and 770 cm^−1^ (imide ring deformation) indicate the presence of imide groups, confirming the formation of imide structures. The disappearance of the absorption peak at 1650 cm^−1^ further confirms the complete imidization of the composite slurry [13]. The composite films with modified AlN exhibit a characteristic absorption band at 2985 cm^−1^ corresponding to the methyl (-CH_3_) groups from the coupling agent, indicating effective modification of the AlN nanoparticles. When the AlN content is less than 15%, only a weak -CH_3_ absorption band is observed due to the low addition of AlN [23].

#### 3.2.4. Thermogravimetric Analysis

**Figure 9:**
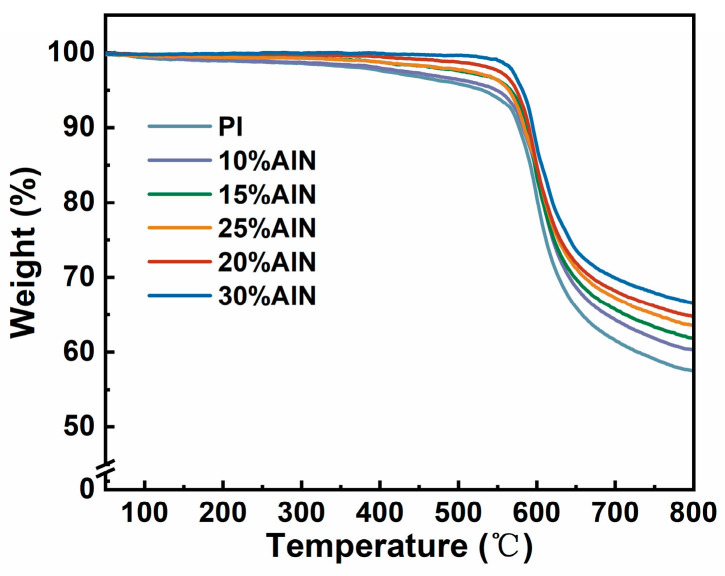
Results of the thermogravimetric.

Thermal stability of the PI/AlN composite films was investigated using thermogravimetric analysis (TGA), and the thermogravimetric curves are shown in Figure 9. The 5 wt% and 10 wt% weight loss decomposition temperatures of the composite films were obtained from the TGA curves, denoted as T_d5%_ and T_d10%_, respectively, and are summarized in Table 1. It is evident that as the concentration of AlN increases, the thermal stability of the composite films improves significantly, demonstrating the positive effect of AlN on enhancing thermal stability. Notably, the T_d10%_ of the composite films exceeded 570 °C, and the 10 wt% thermal decomposition temperature of the 30% AlN-doped composite film reached 588 °C, which indicates excellent thermal resistance. From the TGA curve, the observed trend clearly demonstrates that the incorporation of AlN leads to a notable enhancement in the pyrolysis temperatures of the composite films, thereby indicating a significant improvement in their thermal stability. This is because AlN particles exhibit minimal thermal loss at elevated temperatures. Additionally, the physical barrier effect formed by nano-AlN particles within the PI matrix can suppress the escape of thermal decomposition products, thereby mitigating thermal decomposition [24].

#### 3.2.5. Thermal Properties Analysis

**Figure 10:**
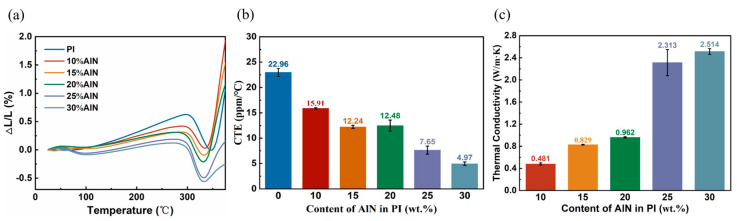
(**a**) TMA curves of composite films; (**b**) dependence of CTE values on AlN content; (**c**) dependence of thermal conductivity on AlN content.

During the wafer bonding process, the materials used are subjected to high-temperature conditions, and it is essential that the filler material possesses a low coefficient of thermal expansion (CTE) to minimize thermal stress with silicon-based materials, thus preventing wafer warpage and interlayer cracking. Furthermore, high thermal conductivity is critical for efficient heat dissipation, preventing overheating of the wafers during high-temperature processing. Figure 10a shows the relationship between the relative change rate and temperature for pure PI films and PI/AlN composite films as measured by a thermomechanical analyzer, from which the CTE values are calculated and presented in Figure 10b. It can be clearly observed that due to the lower CTE of AlN, doping nano-AlN particles into PI effectively reduces the coefficient of thermal expansion of the films.

The modified nano-AlN particles exhibit strong interactions with PI, restricting the thermal motion of PI molecular chains and thereby significantly reducing the CTE of the composite films at high temperatures. Equation (1) is the calculation formula for the CTE. The CTE is commonly denoted as α and expressed in units of 1/K or ppm/K. The first inflection point observed in the CTE curve corresponds to a transition in the properties of the composite film. This temperature was selected as the critical point, and the CTE value was calculated over the temperature range from room temperature to this critical point according to Equation (1). When the AlN doping concentration reaches 30%, the CTE of the composite film can achieve 4.97 ppm/K, closely matching that of silicon, which ensures minimal deformation in high-temperature environments.

(1) $$\alpha =\frac{\Delta L}{L\cdot \Delta T}$$

Figure 10c presents the thermal conductivity of PI/AlN composite films as measured by a laser flash apparatus. Owing to the intrinsically high thermal conductivity of AlN, the incorporation of modified nano-AlN particles into the PI effectively enhances the thermal conductivity of the films. With increasing AlN content, more thermally conductive filler networks are formed within the matrix, promoting the construction of continuous heat conduction pathways. This reduces phonon scattering and facilitates more efficient heat transfer through particle–particle contacts. Moreover, as the filler content further increases, a percolation-like thermal conduction network is progressively established. When the AlN content is increased to 25%, the formation of interconnected pathways becomes more pronounced, resulting in a substantial improvement in thermal conductivity. When the AlN doping concentration reaches 30%, the composite films achieve a thermal conductivity of 2.514 W/m·K.

#### 3.2.6. Mechanical Properties Analysis

**Figure 11:**
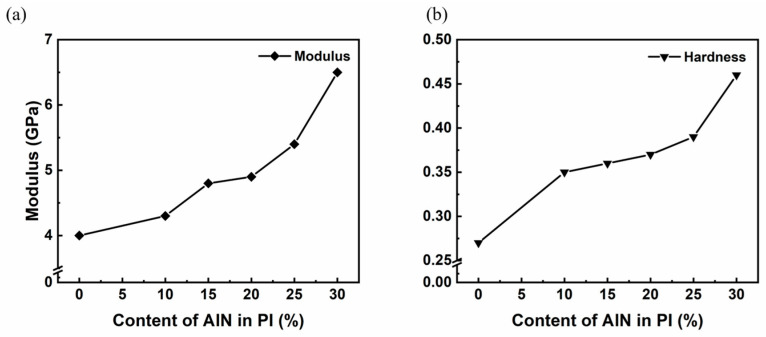
(**a**) Dependence of modulus on AlN content; (**b**) dependence of hardness on AlN content.

Good mechanical properties, particularly resistance to deformation and hardness, are essential for the filler material to prevent deformation or cracking under mechanical stress. The pure PI film exhibits an insufficient elastic modulus and hardness, which may lead to defects such as filling collapse or abrasion. The nano-indentation method combined with a nano-indenter was adopted to characterize the elastic modulus and hardness of pure PI and composite films. The test results of the elastic modulus and hardness of the composite films are shown in Figure 11. It is clearly observed from the figure that both elastic modulus and hardness of the composite films improve significantly with increasing AlN content. Specifically, when the AlN doping concentration reached 30%, the elastic modulus of the composite film reached 6.5 GPa, which is 62.5% high than that of pure PI film (4 GPa). Similarly, the hardness improved from 0.27 GPa to 0.46 GPa, which is increased by 70.4%. However, when the AlN doping content exceeded 30%, the nanoparticles became difficult to uniformly disperse within the high-viscosity PI matrix, negatively impacting the overall stability of the composite films. Additionally, excessively high modulus values could exacerbate thermal stress mismatch, and overly high hardness could lead to brittle fracture, both reducing interfacial reliability [25]. Thus, the elastic modulus and hardness in this study for the composite films are within a balanced condition, effectively enhancing deformation resistance while avoiding issues associated with excessively high mechanical properties. Doping the PI matrix with AlN particles not only optimizes its thermo-mechanical stability but also significantly improves its mechanical properties.

### 3.3. Application in Wafer Channel Filling

#### 3.3.1. Results of Wafer Channel Filling

DIW technology offers advantages such as controllable extrusion temperature, wide material applicability, ease of use, and high precision. Considering filling efficiency, cost, and environmental impact, this study proposes using DIW to fill wafer channels with polymer resin materials.

To observe the filling state of the wafer channels, the filled wafer was cut along the vertical direction of the channels. A cylindrical mold with a diameter of 2 cm was used to prepare samples for SEM observation of the channel cross-section. The wafer with the channel was fixed with the channel cross-section facing the bottom of the mold. Epoxy resin was poured in and left to cure. After curing, the sample was polished to expose the wafer channel, followed by mechanical polishing to smooth the surface. After sample preparation, the wafer channel was subjected to SEM and EDS analysis.

**Figure 12:**
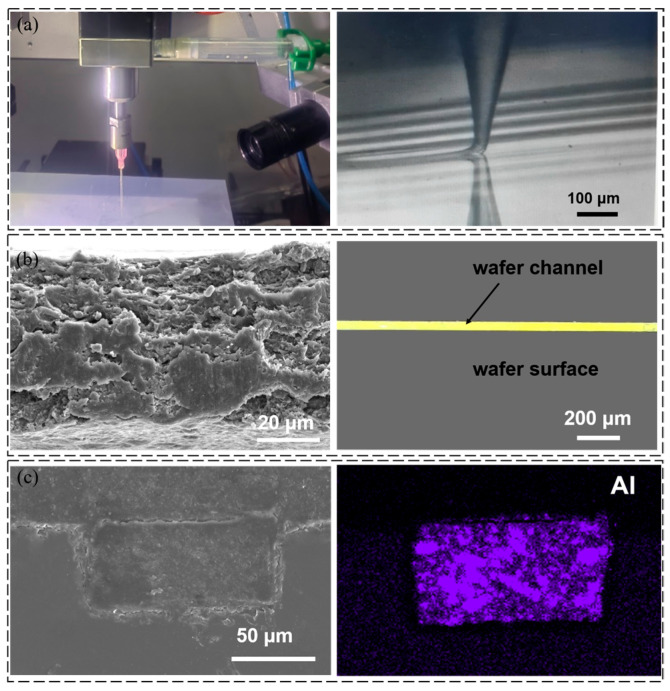
Results of wafer channel filling. (**a**) Custom-built DIW experimental platform and extrusion process of the composite ink; (**b**) cross-sectional SEM images of the film after repeated printing and curing and the surface of the wafer after filling; (**c**) cross-sectional SEM images of the wafer channel after filling and the distribution of Al element.

As shown in Figure 12a, wafer channel filling experiments were conducted using a custom-built DIW experimental platform [10]. To control the flow rate effectively, the composite ink was extruded using pneumatic control. A glass needle with an inner diameter of 30 μm enabled uniform and smooth extrusion of the composite ink. The extrusion pressure (100–200 kPa) and printing speed (5–10 mm/s) were optimized through a parameter screening process, in which different combinations of pressure and speed were systematically tested. The optimal parameter window was identified based on extrusion stability, channel filling completeness, and defect formation, where stable continuous deposition and complete wafer channel filling were simultaneously achieved. The extrusion rate was governed by the applied pneumatic pressure and nozzle geometry. The results of wafer channel filling are presented in Figure 12b,c. After repeating the print-cure cycle three times, the wafer channels were completely filled. Post-filling examination of the wafer surface revealed no overflow, and cross-sectional observation of the repeatedly printed and cured films showed no delamination, thus avoiding stress concentration issues due to interlayer cracking. These findings confirm that DIW technology is a reliable and effective method for wafer channel filling, and the prepared composite ink meets the filling requirements.

#### 3.3.2. Bonding Force Analysis

The bonding force between the thin films and the silicon substrates was investigated using micro-scratch testing with a micro-scratch tester. The test parameters were as follows: scratch rate of 6 mm/min, scratch length of 3 mm, continuous loading mode, load range from 0.01 to 5 N, loading rate of 9.98 N/min, diamond indenter material, and indenter radius of 200 μm.

**Figure 13:**
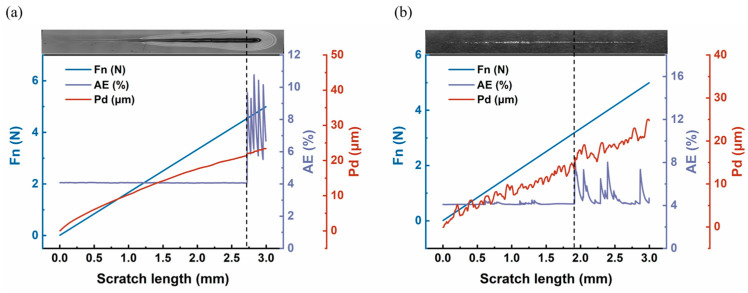
Results of bonding force test. (**a**) PI films and silicon substrate; (**b**) composite film with 30% AlN content and silicon substrate.

The slurry was cured on a silicon wafer, and the interfacial bonding force between the pure PI film and the substrate is shown in Figure 13a. The curve represents the variation in applied load (Fn), acoustic emission (AE) signals, and penetration depth (Pd) as a function of scratch length. Fn corresponds to the continuously applied load, and the curve exhibits linear growth. In the initial stage, the AE signal remained stable, and Pd increased uniformly, indicating a homogeneous internal structure of the PI film. At a scratch length of 2.72 mm, a sudden change occurred in the AE signal, and the signal fluctuated thereafter. The Pd also experienced a sharp change at this point, marking the critical point where the film and substrate experienced failure. The critical load was 4.53 N. Optical microscope images of the scratch revealed noticeable delamination marks around the scratch, which might be due to crack propagation after the film-substrate failure.

Figure 13b shows the bonding force test curve for the 30% AlN-doped composite film and silicon substrate. It is evident that the Pd and AE signal curves experienced a sudden change at the scratch length of 1.91 mm, identifying this point as the critical failure point between the composite film and the substrate, with the corresponding critical load of 3.19 N. Prior to this, slight fluctuations in the AE signal and Pd curve were observed, caused by the presence of AlN particles within the film. The composite film exhibited lower bonding force with the silicon substrate compared with pure PI, due to the latter’s higher uniformity and fewer interfacial defects. Pure PI can form stronger bonds with silicon via van der Waals forces and polar molecular groups [26].

The reduction in adhesion strength may indicate an increased risk of interfacial degradation under long-term thermal cycling conditions due to thermal stress induced by CTE mismatch. However, with 30% AlN doping, the CTE of the composite film is significantly reduced compared with that of pure PI, which helps alleviate the thermal mismatch with the Si substrate. Considering the balance between interfacial adhesion and improved thermomechanical performance, the AlN-doped composite film is still expected to maintain acceptable long-term reliability under normal operating conditions.

## 4. Conclusions

This study presents a method for filling wafer channels and the preparation of corresponding filling materials. It also discusses the effects of varying AlN content on the properties of PI/AlN composites. The key findings are as follows:

1. The AlN/PI composite ink showed stable, uniformly distributed nano-AlN particles and optimal viscosity, ensuring uniform dispersion and precise flow control during the DIW process. It also had a high solid content, enhancing filling efficiency;
2. The composite films exhibited excellent thermal stability. At 30% AlN content, the 10 wt% thermal decomposition temperature was 588 °C, and the CTE decreased significantly, reaching 4.97 ppm/K, better matching that of silicon-based materials, effectively preventing stress concentration during subsequent high-temperature processes;
3. The composite films possessed outstanding mechanical performance. At 30% AlN content, the elastic modulus and hardness reached 6.5 GPa and 0.46 GPa, which increased by 62.5% and 70.4% compared with pure PI film, respectively. The optimized elastic modulus and hardness avoid thermal-stress mismatch and brittle fracture, satisfying the mechanical requirements for wafer channel filling materials.
4. DIW technology efficiently filled wafer channels in three filling cycles, with no surface overflow, delamination, and a high interfacial bonding force, indicating that this method is effective and reliable.

Compared with the conventional PECVD method for wafer channel filling, the DIW technique with composite materials improves filling efficiency and reduces costs. Owing to the CTE match between the PI/AlN composites and the silicon wafer, the whole wafer after channel filling is favorable in the downstream processes such as CMP and reflow, as well as the entire wafer handling process, which reduces the potential cracking risk. This study not only contributes to the enhancement of electronic packaging materials such as polyimide but also holds significant innovative value for heterogeneous integration and wafer-level packaging.

## Figures and Tables

**Table 1:** Degradation temperature of composite films at 5% and 10% weight loss.

	0 wt% (Pure PI)	10 wt%	15 wt%	20 wt%	25 wt%	30 wt%
T_d5%_ (°C)	534	549	567	566	572	576
T_d10%_ (°C)	579	582	588	585	587	588

## Data Availability

The original contributions presented in this study are included in the article. Further inquiries can be directed to the corresponding authors.

## Abbreviations

The following abbreviations are used in this manuscript:

3D HI	three-dimensional heterogeneous integration
C2W	Chip-to-wafer
DIW	Direct ink Writing
PI	Polyimide
AlN	Aluminum nitride
CTE	Coefficient of thermal expansion
PECVD	Plasma-enhanced chemical vapor deposition
CMP	Chemical Mechanical Polishing
